# The birth of piRNAs: how mammalian piRNAs are produced, originated, and evolved

**DOI:** 10.1007/s00335-021-09927-8

**Published:** 2021-11-01

**Authors:** Yu H. Sun, Brent Lee, Xin Zhiguo Li

**Affiliations:** 1 Center for RNA Biology: From Genome to Therapeutics, Department of Biochemistry and Biophysics, Department of Urology, University of Rochester Medical Center, Rochester, NY 14642 USA; 2 Department of Biology, University of Rochester, Rochester, NY 14642 USA

## Abstract

PIWI-interacting RNAs (piRNAs), small noncoding RNAs 24–35 nucleotides long, are essential for animal fertility. They play critical roles in a range of functions, including transposable element suppression, gene expression regulation, imprinting, and viral defense. In mammals, piRNAs are the most abundant small RNAs in adult testes and the only small RNAs that direct epigenetic modification of chromatin in the nucleus. The production of piRNAs is a complex process from transcription to post-transcription, requiring unique machinery often distinct from the biogenesis of other RNAs. In mice, piRNA biogenesis occurs in specialized subcellular locations, involves dynamic developmental regulation, and displays sexual dimorphism. Furthermore, the genomic loci and sequences of piRNAs evolve much more rapidly than most of the genomic regions. Understanding piRNA biogenesis should reveal novel RNA regulations recognizing and processing piRNA precursors and the forces driving the gain and loss of piRNAs during animal evolution. Such findings may provide the basis for the development of engineered piRNAs capable of modulating epigenetic regulation, thereby offering possible single-dose RNA therapy without changing the genomic DNA. In this review, we focus on the biogenesis of piRNAs in mammalian adult testes that are derived from long non-coding RNAs. Although piRNA biogenesis is believed to be evolutionarily conserved from fruit flies to humans, recent studies argue for the existence of diverse, mammalian-specific RNA-processing pathways that convert precursor RNAs into piRNAs, perhaps associated with the unique features of mammalian piRNAs or germ cell development. We end with the discussion of major questions in the field, including substrate recognition and the birth of new piRNAs.

## Introduction

**Fig. 1:**
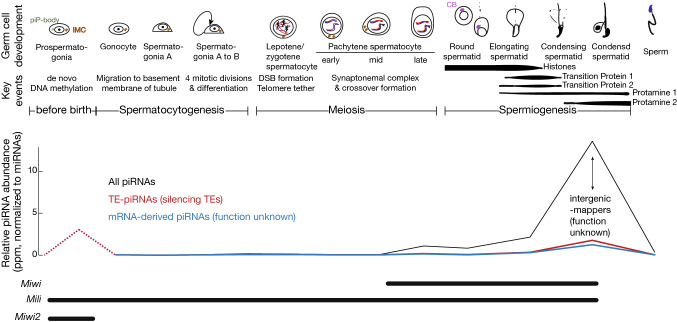
Key events in mouse spermatogenesis and their associated piRNA and PIWI gene expression. The top two panels show germ cell development stages and corresponding key events. The third panel from the top shows piRNA expression levels. piRNA abundance is measured by small RNA sequencing based on (Li et al. 2013) and unpublished data. The bottom panel shows the expression profiles of three PIWI genes in mice: *Miwi* *Mili* and *Miwi2*, *DSB* Double-strand break, *TE* transposable element. Dash line represents putative data, *IMC* intermitochondrial cement, *CB* chromatoid body

PIWI-interacting RNAs (piRNAs) are small non-coding RNAs that form 1:1 RNA–protein complexes with PIWI (P-element-induced wimpy testis) proteins. The PIWI gene was first identified in 1997, the disruption of which leads to defects in germ stem cell maintenance in *Drosophila* (Lin and Spradling 1997). Further studies revealed that a conserved family of PIWI genes with an essential function in germ cells is widely distributed in both vertebrates and invertebrates (Chirn et al. 2015; Murchison et al. 2008; Wynant et al. 2017). Three PIWI paralogs are found in mice, MIWI, MILI, and MIWI2, the deletion of any one of which leads to male infertility (Aravin et al. 2007b; Deng and Lin 2002; Carmell et al. 2007) (Fig. 1). piRNAs were first reported in 2001 in *Drosophila* as small silencing RNAs with distinct features from known microRNAs (miRNAs) or small interference RNAs (siRNAs) (Aravin et al. 2001). Although research in Tetrahymena in 2002 indicated that PIWI proteins bind small RNAs and the RNA–protein complex functions together in genome rearrangement (Box 1), the name “piRNA” was not coined until a group of studies published in 2006 revealed that a set of germ cell-specific small RNAs associate with PIWI proteins in both *Drosophila* and mammals(Vagin et al. 2006; Girard et al. 2006; Aravin et al. 2006; Grivna et al. 2006; Lau et al. 2006). These studies uncovered the features that distinguish piRNAs from other small RNAs: (1) piRNAs have a characteristic length of 24–35 nucleotides (nt), which is longer than both miRNAs and endogenous siRNAs; (2) 2′-*O*-methyl modifications occur at the 3′-end of piRNAs; and (3) piRNAs associate with PIWI proteins, a subclade of Argonaute proteins. While later studies have confirmed the distribution of piRNAs across bilateral animals (Grimson et al. 2008; Lewis et al. 2018), and piRNA biogenesis is believed to be evolutionarily conserved from fruit flies to humans (Czech and Hannon 2016a; Gainetdinov et al. 2018), recent studies argue for the existence of diverse, mammalian-specific RNA-processing pathways that convert precursor RNAs into piRNAs, probably associated with the unique features of mammalian piRNAs or germ cell development. Therefore, this review focuses primarily on mouse piRNAs, although piRNAs from other organisms will be discussed when relevant.

## Multi-faceted piRNAs—classifying piRNAs

In mice, piRNAs are divided into three major classes based on their origins (Table 1): transposable element (TE) piRNAs from DNA transposons, endogenous retroviruses, long interspersed nuclear elements (LINEs), or short interspersed nuclear elements (SINEs); intergenic piRNAs from non-coding regions of the genome; and 3′untranslated region (3′UTR) piRNAs that map to the 3´UTRs of protein-coding regions in the sense orientation. TE piRNAs protect the germline genome from TE activation, a function that is evolutionarily conserved in bilateral animals (Kumar and Carmichael 1998; Aravin and Hannon 2008; Farazi et al. 2008; Thomson and Lin 2009; Grimson et al. 2008). TE piRNAs are the dominant piRNA class in *Drosophila* and zebrafish, whereas intergenic piRNAs are the dominant piRNA class in adult mammalian testes. Both 3′UTR piRNAs and intergenic piRNAs are mostly uniquely mapped to the genome and lack well-identified targets. A small fraction of intergenic piRNAs have been shown to base-pair with and trigger the decay of mRNAs required for sperm maturation (Goh et al. 2015; Gou et al. 2014; Zhang et al. 2015; Wu et al. 2020). 3′UTR piRNAs have been detected in *Drosophila*, frogs, and diverse mammalian species (Robine et al. 2009; Chirn et al. 2015). These 3′UTR piRNAs are derived from full-length mRNA precursors rather than cryptic transcripts corresponding exclusively to 3′UTRs, and their biogenesis is coupled with translation thus fine-tuning protein synthesis from their mRNA precursors (Sun et al. 2021).

**Table 1:** Classification of piRNAs in mammals

Precursors	TE RNA	lncRNA	3′UTR RNA
Developmental stage	Pre-natal	Pachytene	Pre-pachytene hybrid
Present	Bilateral animals	Mammals	*Drosophila* to mice
Function	Silence TEs	Silence mRNA	Fine-tune protein synthesis
Biogenesis	Ping-Pong, phased biogenesis	Phased biogenesis, TDRD5 (Tudor Domain Containing 5), and ribosomes	Phased biogenesis, TDRD5, and ribosomes

piRNAs can be categorized into primary piRNAs and secondary piRNAs based on their biogenesis pathways, which are principally distinguished by their mechanisms of 5′end formation. Primary piRNAs are produced from long single-stranded RNA precursors that are synthesized from piRNA loci, and their 5′ends are produced during the fragmentation process, likely involving the MitoPLD endonuclease (or PLD6, which has a homolog “Zucchini” in *Drosophila*) located on mitochondrial outer membranes. Secondary piRNAs are produced from piRNA-targeted transcripts, and their 5′ends are generated by the endonucleolytic activity of PIWI proteins, which cleaves between positions 10 and 11 of the base-pair complementary RNA target relative to the piRNA 5′end. These secondary piRNAs can target the primary piRNA precursor transcripts to generate more secondary piRNAs, resulting in a piRNA-specific “Ping-Pong” loop. This loop is believed to represent an adaptive immune response that enables piRNAs to silence TE transcripts post-transcriptionally, as the loop continues to produce TE piRNAs until the TE transcripts are diminished (Gunawardane et al. 2007; Brennecke et al. 2007). In *Drosophila*, secondary piRNAs can also trigger a Zucchini-dependent “phased” piRNA biogenic mechanism that resembles primary piRNA biogenesis but only occurs downstream of the initial PIWI-catalyzed cleavage event in a 5′-to-3′ stepwise manner, generating non-overlapping fragments known as pre-piRNAs. After loading onto PIWI proteins, the pre-piRNAs (23–42 nt) are further trimmed and methylated to become mature piRNAs (Mohn et al. 2015; Han et al. 2015; Homolka et al. 2015; Ding et al. 2017; Gainetdinov et al. 2018; Darricarrère et al. 2013; Ishizu et al. 2015).

Based on the dynamics of their expression, mouse piRNAs can be further classified into prenatal piRNAs, pre-pachytene piRNAs, pachytene piRNAs, and hybrid piRNAs. Prenatal piRNAs are TE-rich and associated with MILI and MIWI2 proteins. MILI-bound prenatal piRNAs silence piRNA target transcripts in the cytosol. MIWI2-bound prenatal piRNAs are shuttled to the nucleus to recruit epigenetic machinery to direct the DNA methylation (Schöpp et al. 2020) of TEs around 13.5–15.5 days post coitum (dpc) (Aravin et al. 2008; Kuramochi-Miyagawa et al. 2008). Knockout of MILI or MIWI2 leads to TE desilencing; however, the catalytic activity of MIWI2 is not required(De Fazio et al. 2011), indicating that MILI-bound piRNAs are sufficient to trigger a robust cytosolic Ping-Pong reaction and the formation of MIWI2-bound secondary piRNAs. Thus, the current data suggest that a subset of MILI cleavage products is loaded to MILI staying in the cytosol to trigger a Ping-Pong loop and another subset of cleavage products is loaded to MIWI2, and the MILI cleavage also triggers downstream phased piRNA production loading to MIWI2 (Yang et al. 2016). Pre-pachytene piRNAs, expressed after birth, have the lowest abundance among the four groups and are composed of TE piRNAs and 3′UTR piRNAs. They are associated with MILI and are essential for silencing TEs during the mitotic and meiotic stages of adult spermatogenesis (Di Giacomo et al. 2013). Pachytene piRNAs, along with MIWI proteins, are produced during the pachytene stage of meiosis. They, associated with both MILI and MIWI, are generally poor in TE complementary sequences and are mostly derived from long non-coding RNA (lncRNA) precursors synthesized in intergenic regions. Pachytene piRNAs represent the most abundant class of small RNAs in the adult testis, around 5.7 to 7.2 μM per cell (Gainetdinov et al. 2018). Pachytene piRNAs are produced via a Ping-Pong independent mechanism (Beyret et al. 2012), and, indeed, MIWI endonucleolytic cleavage activity is not required for pachytene piRNA production. Hybrid piRNAs are a class of piRNAs with features of both pachytene piRNAs (being present at increased levels during the pachytene stage and derived from lncRNAs) and pre-pachytene piRNAs (which map to mRNA 3′UTRs) (Li et al. 2013). Hybrid piRNA activation during pachytene stage is driven by the same transcription factor A-MYB as pachytene piRNAs, and their 3′UTRs are embedded with TE sequences, thus producing TE piRNAs that trigger Ping-Pong loops silencing TEs (Sun et al. 2021).

## Transcription of piRNA loci

**Fig. 2:**
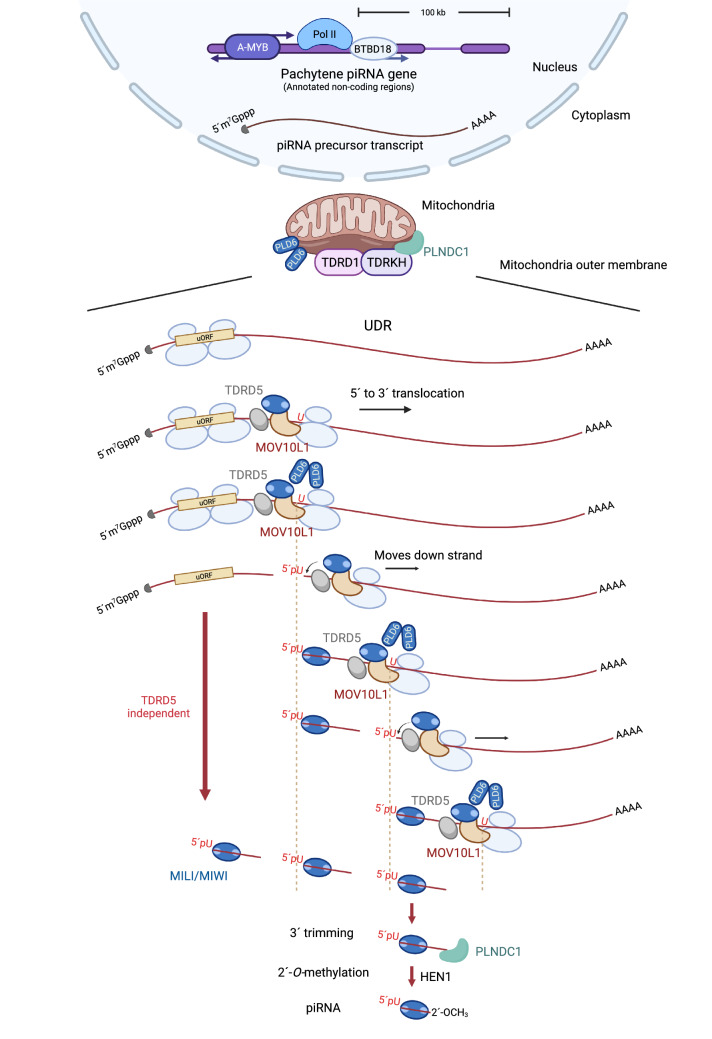
Current model of pachytene piRNA biogenesis in mouse testis. Facilitated by A-MYB and BTBD18, pachytene piRNA precursor transcripts are synthesized by RNA polymerase II, containing a 5′-cap and a poly(A) tail. Introns of these precursors are spliced out, and then these precursors are transported from the nucleus to the cytoplasm and further located to the IMC. Presumably, endonuclease PLD6 on the outer membrane of mitochondria cleaves the piRNA precursors and generates the 5′ ends of future piRNAs. In the first phase of piRNA biogenesis, ribosomes translate the uORF region and piRNAs are produced in a TDRD5-independent manner. In the second phase, ribosomes translocate to the UDR region, facilitated by MOV10L1, and guide piRNA production in a TDRD5-dependent manner. Finally, these cleaved products will be loaded onto MILI or MIWI protein for further 3′ end maturations that PNLDC1 trims and HEN1 adds the 2′-*O*-methyl group to the end of piRNAs. Moreover, MOV10L1 and TDRD5 bind directly to pachytene piRNA precursors. Figure created with BioRender.com

Despite piRNAs being the most heterogenous small non-coding RNA in animals with > 1 million unique sequences detected in individual germlines from most animal species that have been studied (Vagin et al. 2006; Aravin et al. 2006; Lewis et al. 2018), they are produced from discrete genomic loci, historically defined using computational methods and named piRNA clusters (Brennecke et al. 2007; Gainetdinov et al. 2018). Later work has defined the transcriptional start sites, polyA cleavage sites, promoters, and splice sites of the transcriptional units in the piRNA loci in mice (Li et al. 2013). Among all classes of murine piRNAs, we understand the transcriptional regulation of pachytene piRNAs in the most detail. Unlike convergent transcribed dual-stranded piRNA loci in *Drosophila* and chickens, piRNA loci in mammals are unidirectionally or bidirectionally transcribed similar to the transcription of protein-coding genes (Gould et al. 2012; Sun et al. 2017; Li et al. 2013; Yu et al. 2021). The single-strand pachytene piRNA precursors, ranging from 500 to 80,000 nt (Betel et al. 2007; Li et al. 2013), are generated with the activation of the transcription factor A-MYB (Li et al. 2013) (Fig. 2). A-MYB-mediated transcriptional regulation of pachytene piRNAs is conserved in amniotes (Li et al. 2013), and A-MYB also regulates the mRNAs coding for piRNA biogenic proteins, forming a feedforward loop to ensure the robust activation of piRNA production during pachytene stage. Unlike promoters from protein-coding genes, which often have a high CpG content, the promoters of pachytene piRNA loci have a low CpG content and are heavily methylated (Yu et al. 2021). RNA polymerase II transcribes these pachytene piRNA genes, and the RNAs undergo conventional mRNA processing, including 5′-capping and polyA tailing. Pachytene piRNA precursors often contain introns that are removed by splicing, indicating that splicing occurs during piRNA precursor synthesis. Those precursors that harbor a long first exon require an additional biogenic factor, BTBD18 (BTB Domain Containing 18), to facilitate their transcriptional elongation (Zhou et al. 2017; Yu et al. 2021). Pachytene piRNA precursors also bind THOC1 and THOC2, THO complex subunits, for their nuclear export (Yu et al. 2021) and are eventually localized to the surface of mitochondria for primary piRNA biogenesis (Li et al. 2013; Murano et al. 2019; Fabry et al. 2019) (Fig. 2).

## Post-transcriptional processing of piRNA precursors

The post-transcriptional processing of all primary piRNA precursors can be simplified to three steps: 5′end formation, PIWI loading, and 3′end formation (Fig. 2). The 5′ends of primary piRNAs are formed when piRNA precursors are fragmented, likely by the MitoPLD endonuclease in mice based on structural and biochemical studies on its homologs in *Drosophila *(Gao and Frohman 2012; Kabayama et al. 2017) and silkworm (Izumi et al. 2020). In mice, MitoPLD, previously shown to be a phospholipid-hydrolyzing enzyme, contains an N-terminal mitochondrial targeting signal and is located on the outer membrane of mitochondria (Gao and Frohman 2012; Kabayama et al. 2017). However, structural studies of *Drosophila* ZUCCHINI suggests that its active site resembles the bacterial endonuclease Nuc, and *Zucchini* displays endonuclease activities in vivo (Voigt et al. 2012). During *Drosophila* phased piRNA biogenesis, ZUCCHINI continuously cleaves the single-stranded RNAs and simultaneously generates the 3′end of the pre-piRNA (before 3′-trimming occurs) and the 5′-end of the next, immediately adjacent pre-pre-piRNA. The cleaved piRNA intermediates, pre-pre-piRNAs, are loaded onto PIWI proteins to trigger the next ZUCCHINI-dependent cleavages between the 3′end of the pre-piRNAs and 5′end of the pre-pre-piRNAs. Recently, the cleavage of single-stranded RNAs by silkworm ZUCCHINI has been recapitulated in vivo (Izumi et al. 2020). MOV10L1 is an RNA helicase required for piRNA biogenesis. It interacts with PIWI proteins stably, while its interaction with pachytene piRNA precursors is transient, requiring crosslink to be detected (Zheng et al. 2010; Vourekas et al. 2015). MOV10L1 is thought to load PIWI proteins onto the pre-pre-piRNAs. The 3′ends of pre-piRNAs undergo trimming by an exonuclease (Trimmer in *Drosophila*, PNLDC1 in mice) and 2′-*O*-methylation by HENMT1 (Hayashi et al. 2016; Kirino and Mourelatos 2007; Ohara et al. 2007).

## Substrate recognition of piRNA precursors

The mechanisms that identify an RNA for piRNA processing in mice are currently unknown. As 5′end formation is upstream of PIWI loading and 3′end formation, the main question is what specifies the initial cleavages on the piRNA precursors. Based on the current model from *Drosophila*, the initiation of ZUCCHINI-dependent phased piRNA processing requires an initiator piRNA that has base-pair complementarity to the piRNA precursors. In *Drosophila*, the initiator piRNAs are provided maternally to the eggs in the germplasm and the cells with germplasm differentiate into germ cells. The maternally deposited initiator RNAs are the secondary piRNAs of the previous (F0) generation, which target the primary piRNA precursors in the germ cells of F1 generation and initiate primary piRNA production in a phased manner. The secondary piRNAs generated from the primary piRNAs will be deposited for primary piRNA production in the following generation (F2) and so on. However, unlike *Drosophila* germ cells, whose fates are pre-determined by the presence of germplasm where the piRNAs are deposited (Santos and Lehmann 2004), mammalian germ cells are induced de novo from somatic cells during embryogenesis (Nicholls et al. 2019). Thus, the biogenesis of pre-natal piRNAs in mice must have been initiated de novo. Similarly, de novo phased piRNA biogenesis through unknown mechanisms occurs in somatic cells of *Drosophila,* where piRNAs do not display Ping-Pong signatures (Homolka et al. 2015). Although it has been proposed that pre-pachytene piRNAs could serve as initiator piRNAs for pachytene piRNA biogenesis (Gainetdinov et al. 2018; Czech and Hannon 2016a), PIWI protein is below the detection limit in spermatocytes before pachytene piRNA production commences (Di Giacomo et al. 2013), arguing against the existence of abundant initiator piRNAs for pachytene piRNA biogenesis being provided from the pre-pachytene stage. Thus, it remains unclear whether the phased biogenesis of pachytene piRNAs is initiated by initiator piRNAs or by a de novo mechanism.

Other than the presence or source of initiator piRNAs, two major biogenic differences between mouse pachytene piRNAs and *Drosophila* germline piRNAs lie with their precursors. First, mouse pachytene piRNA precursors are long and continuous transcripts. Although both types of precursors are single-stranded RNAs, *Drosophila* germline piRNA precursors are cryptic transcripts that are synthesized in a heterochromatin-dependent and promoter-independent mechanism (Andersen et al. 2017), whereas pachytene piRNA precursors have defined transcriptional initiation sites and polyA cleavage sites and are long continuous RNAs up to 80,000 nt in length. *Drosophila* germline piRNA precursors are derived from highly repetitive regions, whereas mouse pachytene piRNA precursors are depleted of TE sequences in comparison to the rest of the genome. Given that downstream phased piRNA production declines with distance (Mohn et al. 2015; Han et al. 2015), which may be due to the instability of cleavage products prior to PIWI loading, more frequent cleavage promoted by initiator piRNAs would be required to produce more phased piRNAs in *Drosophila *(Mohn et al. 2015). However, the lack of repetitive elements and low abundance of piRNA prior to the pachytene stage argue against frequent initiator piRNA-mediated cleavage on pachytene piRNA precursors. Because of the low number of cleavages, if any, promoted by initiator piRNAs, phased pachytene piRNA biogenesis must be highly processive, as single-stranded RNAs are known to form secondary structures and bind to RNA-binding proteins. This requirement for high processivity of phased production may explain why ribosomes are involved in piRNA biogenesis in vertebrates, as elongating ribosomes are strong helicases (Takyar et al. 2005; Qu et al. 2011), unwinding secondary structures or stripping RNA-binding proteins off the precursors.

Second, mouse pachytene piRNA precursors are translated. In *Drosophila,* the RNA granules where piRNA precursors are processed are in close proximity to the nucleus, allowing direct channeling of the precursors. However, such attachment between the nucleus and RNA granules is not observed in mice. This may cause increased dwell time for piRNA precursors in the cytosol and can promote ribosome translation of the upstream open reading frame (uORF) of piRNA precursors, which are nonetheless annotated as lncRNAs (without long or conserved ORFs) (Sun et al. 2020). After translation of the uORFs, MOV10L1 facilitates the translocation of 80S ribosomes into the uORF downstream regions that produce the majority of piRNAs (Yabuta et al. 2011; Zheng and Wang 2012; Sun et al. 2018; Ding et al. 2018; Guan et al. 2021). Endonucleolytic cleavage occurs on ribosome-bound piRNA precursors near the ribosome E site, generating the pre-pre-piRNAs with ribosomes bound at their 5′-extremities(Sun et al. 2020) (Fig. 2). Given that ribosomes are actively moving along the UDR, based on runoff assays after translation inhibition, the current model proposes that once the ribosomes translocate downstream, PIWI proteins are loaded onto the 5′P end of pre-pre-piRNAs and trigger MitoPLD-dependent cleavage between the ribosomes and PIWI proteins. Although this ribosome-guided piRNA biogenesis mechanism is detected in chickens and lizards, it does not appear to exist in invertebrates (Izumi et al. 2020), nor does it function for regions of uORFs and 5′UTRs of pachytene piRNA precursors in mice. The phenotype of *Tdrd5* mutant mice demonstrates the distinct biogenesis pathways that operate 5′ (upstream) and 3′ (downstream) of the stop codon of uORFs, as only the production of piRNAs from uORF downstream regions requires TDRD5, a Tudor domain protein that binds to pachytene piRNA precursors (Ding et al. 2018) (Fig. 2). Thus, it is possible that the ribosome-guided, TDRD5-dependent mechanism has specifically evolved for long continuous piRNA precursors. The differences between mice and *Drosophila* piRNA precursors suggest that more mammalian-specific biogenic factors involved in substrate recognition and coordination of substrate processing with ribosome translocation have yet to be discovered.

## Summary of piRNA biogenic factors

Mouse piRNA biogenic factors, along with their homologs in *Drosophila*, include transcription factors (A-MYB, BTBD18), PIWI proteins (MIWI2, MILI, MIWI), multiple Tudor domain-containing proteins, ribosomes, endonucleases (MitoPLD), 3′end maturation enzymes (PNLDC1, HENMT1), and other co-factors (MOV10L1, DDX4, GTSF1, MAEL, etc.) (Table 2). Some factors perform functions that are highly conserved between *Drosophila* to mammalians, such as MitoPLD (PLD6, or Zucchini in *Drosophila*) and S-adenosylmethionine (SAM)-dependent methyltransferase (Hen1 in *Drosophila*; HENMT1 in mice) (Table 2). On the other hand, some factors, representing homologous proteins between *Drosophila* and mice, have gained novel functions through gene duplication, such as MOV10 and MOV10L1, both of which are mouse homologs of *Drosophila Armitage*. A-MYB, BTBD18, and ribosomes represent factors specific to amniote (including mammals and birds) piRNA biogenesis (Li et al. 2013; Zhou et al. 2017; Sun et al. 2020). Tudor domain-containing proteins (Gan et al. 2019) are essential for piRNA biogenesis in both *Drosophila* and mice, including TDRD1, TDRD2 (TDRKH), TDRD4 (RNF17), TDRD5, TDRD6, TDRD7, TDRD9, and TDRD12 (ECAT8) (Table 2). The 3′end trimming protein in mice is a poly(A)-specific ribonuclease-like domain-containing 1 (PNLDC1), an ortholog protein missing in *Drosophila* but present as PARN-1 in *C. elegans* and silkworm. *Drosophila* instead uses the miRNA-trimming enzyme Nibbler to shorten piRNAs (Liu et al. 2011; Han et al. 2011; Feltzin et al. 2015), indicating the existence of diverse mechanisms for piRNA 3′end formation (Czech and Hannon 2016b).

**Table 2:** piRNA biogenesis factors in mice

Mouse	*Drosophila*	Function
A-MYB (MYBL1)	Myb	A-MYB is a transcription factor of mouse pachytene piRNAs. It binds to the promoter regions and drives the transcription of both pachytene piRNA precursors, hybrid piRNA precursors, and the mRNAs for core piRNA biogenesis factors including MIWI(Sun et al. 2021; Li et al. 2013)
BTBD18	–	As a nuclear protein, BTBD18 occupies a subset of pachytene piRNA-producing loci and facilitates their transcriptional elongation mediated by RNA polymerase II(Zhou et al. 2017)
MIWI (PIWIL1)	Aubergine (Aub)	Pachytene-expressed MIWI binds to piRNAs with a typical length of ~ 29–31 nt. In adult mouse testes, piRNAs are loaded to MILI and MIWI. MIWI’s PIWI domain has slicer activity which is responsible for TE silencing(Deng and Lin, 2002; Reuter et al. 2011)
MILI (PIWIL2)	Aubergine (Aub)	In primordial germ cells, mouse MILI forms Ping-Pong with MIWI2 to silence TEs. In adult mouse testes, piRNAs are loaded to MILI and MIWI. MILI’s PIWI domain has slicer activity which is responsible for TE silencing(De Fazio et al. 2011; Kuramochi-Miyagawa et al. 2008)
MIWI2 (PIWIL4)	Piwi	In primordial germ cells, mouse MILI forms a Ping-Pong loop with MIWI2 to silence transposons. MIWI2 performs transcriptional silencing with nuclear function(Aravin et al. 2008; Kuramochi-Miyagawa et al. 2008; Darricarrère et al. 2013; Carmo-Fonseca et al. 1991)
GTSF1 (CUE110)	Asterix (Arx)	GTSF1 is an essential factor for secondary piRNA production through MILI-MIWI2 Ping-Pong amplification in mice(Yoshimura et al. 2009, 2018)
MAEL	Maelstrom (Mael)	MAEL localizes predominantly at perinuclear nuage of mouse spermatocytes with a small subset clustered at nucleus and nuclear pores, and is critical for transcriptional repression of TEs(Matsumoto et al. 2015; Findley et al. 2003; Aravin et al. 2009; Sienski et al. 2012; Soper et al. 2008; Castaneda et al. 2014)
MitoPLD (PLD6)	Zucchini (Zuc)	MitoPLD is a mitochondrial outer membrane protein with endonuclease activity. It cleaves the piRNA precursors, which is required to generate the 5′-end of mature piRNAs. So far, no sequence specificity has been reported for MitoPLD’s cleavage activity in vitro(Haase, 2016; Nishimasu et al. 2012; Ipsaro et al. 2012)
GPAT2	Minotaur (Mino)	GPAT2 is a structural component of IMC required for phased piRNA biogenesis. It binds to MILI and plays a critical role in primary processing during piRNA production(Shiromoto et al. 2013; Vagin et al. 2013)
GASZ (ASZ1)	Gasz (CG2183)	GASZ is a mitochondrial outer membrane protein required for phased piRNA biogenesis. It is involved in the silencing of retrotransposons by stabilizing MILI in the nuage(Czech et al. 2013; Ma et al. 2009)
MOV10L1	Armitage (Armi)	MOV10L1 is an RNA helicase required for phased piRNA biogenesis. It unwinds secondary structures such as G quadruplexes on piRNA precursors and promotes phased piRNA biogenesis by facilitating ribosome binding on the uORF downstream regions (UDRs) (Zheng et al. 2010; Frost et al. 2010; Vourekas et al. 2015)
MVH (mouse VASA homolog, DDX4)	Vasa (Vas)	MVH is a DEAD box containing protein with ATP-dependent RNA helicase activity. It is required for the handover of PIWI-cleaved piRNA intermediates, which allows successful secondary piRNA biogenesis(Wenda et al. 2017; Kuramochi-Miyagawa et al. 2010; Xiol et al. 2014)
TDRD1	Vreteno (Vret)	TDRD1 is a Tudor domain-containing protein. In mice, it is localized in the nuage (intermitochondrial cement and chromatoid body) of mouse germ cells and binds to the arginine-methylated MILI protein, serving as a scaffold for piRNA biogenesis(Chuma et al. 2006). In *Drosophila*, it is localized to the nuage and Yb body(Zamparini et al. 2011)
TDRKH (TDRD2)	Papi	TDRKH is a Tudor domain-containing mitochondrial protein involved in piRNA 3′ end processing by tethering PNLDC1 to the mitochondria. It is required for primary piRNA biogenesis but not for the Ping-Pong cycle. It partners with MIWI and MIWI2 via symmetrically dimethylated arginine residues(Chen et al. 2009; Ding et al. 2019; Saxe et al. 2013; Honda et al. 2013)
RNF17 (TDRD4)	Qin	RNF17 is a Tudor domain-containing protein. In mouse testes, RNF17 blocks promiscuous activity of PIWI proteins, and RNF17 mutants show inappropriate Ping-Pong targeting protein-coding genes and long noncoding RNAs. In *Drosophila*, it is localized to the nuage and suppresses homotypic Aub:Aub Ping-Pong(Wasik et al. 2015; Zhang et al. 2014; Pan et al. 2005)
TDRD5	Tejas (Tej)	TDRD5 is a Tudor domain-containing protein localized to the nuage. It is required for UDR piRNA biogenesis in mice but dispensable for piRNA production at the uORF region(Smith et al. 2004; Yabuta et al. 2011; Ding et al. 2018)
TDRD6	Tudor	TDRD6 is a Tudor domain-containing protein. It interacts with MILI and MIWI and is critical for the architecture of chromatoid bodies in mice(Nishida et al. 2009; Vasileva et al. 2009)
TDRD7	Tapas	TDRD7 is a Tudor domain-containing protein. It is localized to the nuage and functions together with TDRD6 in the initial assembly of chromatoid bodies in mice(Patil et al. 2014; Tanaka et al. 2011; Hosokawa et al. 2007)
TDRD9	Spindle-E (Spn-E)	TDRD9 is a Tudor domain-containing protein. It is localized to the nuage (piP body and chromatoid body) and required for Ping-Pong(Wenda et al. 2017; Shoji et al. 2009)
TDRD12 (ECAT8)	Yb, Brother of Yb (BoYb), and Sister of Yb (SoYb)	TDRD12 is a Tudor domain-containing protein. It does not co-localize with DDX4, but it is co-localized to an acrosome structure protein lectin-PNA in round spermatids. It is required for MIWI2-bound secondary piRNA formation(Handler et al. 2011; Pandey et al. 2013; Kim et al. 2016). Its function is facilitated by its interaction partner Exonuclease domain-containing 1 (EXD1)(Pandey et al. 2018)
Ribosome	-	Ribosomes guide the 5′ end formation of pachytene piRNAs in mouse germ cells, mainly from the UDRs of pachytene piRNA precursors, and may act as a strong helicase to sustain phased biogenesis(Mao and Qian 2020; Sun et al. 2021, 2020)
FKBP6	Shutdown (Shu)	FKBP6 is a co-chaperone protein functioning with HSP90 to facilitate piRNA loading onto MIWI2(Olivieri et al. 2012; Preall et al. 2012; Xiol et al. 2012)
HSP90	Hsp83	HSP90 is a co-chaperone protein functioning with FKBP6 to facilitate piRNA loading onto MIWI2(Specchia et al. 2010; Xiol et al. 2012; Olivieri et al. 2012)
PNLDC1	Nibbler*	PNLDC1 is the pre-piRNA 3′ trimming exonuclease which shortens the pre-piRNA 3′ end to allow it to fit into the PIWI protein(Feltzin et al. 2015; Liu et al. 2011; Han et al. 2011; Nishimura et al. 2018; Zhang et al. 2017; Ding et al. 2017)
HENMT1	Hen1	HENMT1 is an S-adenosylmethionine (SAM)-dependent methyltransferase that catalyzes 2′-*O*-methylation formation at the piRNA 3′ end(Lim et al. 2015; Saito et al. 2007; Wang et al. 2016; Hayashi et al. 2016; Kirino and Mourelatos, 2007; Ohara et al. 2007; Horwich et al. 2007)

^*^Nibbler is not an ortholog of PNLDC1 but also trims the pre-piRNAs from their 3′ ends

## Location for piRNA biogenesis and function: RNA granules

Key piRNA biogenic factors, such as PNLDC1, TDRKH, MitoPLD, GASZ, and GPAT2, are found on the outer membrane of mitochondria (Honda et al. 2013; Czech et al. 2013; Ma et al. 2009; Shiromoto et al. 2013; Vagin et al. 2013; Nishimasu et al. 2012; Ipsaro et al. 2012; Haase et al. 2010) where specific RNA granules are located between adjacent mitochondria, called intermitochondrial cement (IMC). A more general term for these RNA- and protein-rich structures in germ cells are germinal granules (or germline granules, or germ granules) (Meikar et al. 2011). These germinal granules are also referred to as a “nuage” (French for “cloud”) due to their amorphous shape, the absence of surrounding membranes, and the abundance of RNA and proteins (Nishida et al. 2007; Seto et al. 2007; Harris and Macdonald 2001; Gunawardane et al. 2007; Brennecke et al. 2007; Meikar et al. 2011). So far, nuages have been found in the germ cells of both vertebrates and invertebrates (Eddy 1975). Nuage morphology, localization, and/or biochemical properties change as germ cells develop (Eddy 1974, 1975; Aravin et al. 2009; Chuma et al. 2009). In mammals, before birth, two types of germinal granules exist: IMC and piP-bodies (Aravin et al. 2009). piP-bodies harbor MIWI2, TDRD9, and MAEL, which are required for secondary piRNAs that will shuttle from the cytosol to nucleus for de novo DNA methylation. piP-bodies are lost after birth, but IMC structures remain until the pachytene stage. Multiple proteins are associated with IMC structures, including MILI, MIWI, TDRD1, TDRD6, TDRD7, TDRD9, MVH (DDX4), and MAEL (Meikar et al. 2011; Castaneda et al. 2014; Soper et al. 2008; Sienski et al. 2012; Aravin et al. 2009; Findley et al. 2003; Matsumoto et al. 2015; Kuramochi-Miyagawa et al. 2010; Xiol et al. 2014; Wenda et al. 2017; Shoji et al. 2009; Tanaka et al. 2011; Patil et al. 2014; Hosokawa et al. 2007; Vasileva et al. 2009; Nishida et al. 2009; Zamparini et al. 2011; Kuramochi-Miyagawa et al. 2004; Deng and Lin 2002). During early pachytene stage, pachytene piRNA precursors are transported to the IMC for piRNA processing. In late pachytene stage, the IMC disappears, and its components diffuse throughout the cytosol (Onohara and Yokota 2012). However, soon after meiosis the components from previous IMC structures aggregate into a single large (∼1 μm) perinuclear granule called a chromatoid body (CB) in haploid round spermatids, which can be clearly observed under the microscope (Meikar et al. 2011).

The CB is recognized by immunofluorescence staining using MVH (DDX4), MILI, and MIWI (Kotaja and Sassone-Corsi 2007; Wang et al. 2009; Meikar et al. 2014, 2011). During spermiogenesis, the CB is initially located near the acrosome and is closely associated with the nuclear envelope (Fawcett et al. 1970). The CB then migrates from the acrosomal region to the caudal pole on the other side of the cell, where it dissociates from the nuclear envelope and remains near the newly grown flagellum (Fawcett et al. 1970). As the sperm flagellum grows, the CB forms a ring structure close to the annulus of flagellum and moves with the annulus, encircling the flagellum (Parvinen 2005; Fawcett et al. 1970). During this process, the CB gradually decreases in size and undergoes progressive disaggregation (Fawcett et al. 1970). In addition to piRNA pathway proteins, the CB also harbors machinery for the miRNA pathway and non-sense mediated mRNA-decay (NMD) pathway (Kotaja and Sassone-Corsi 2007). In sum, for the three types of germinal granules, we propose that IMC is specific for piRNA biogenesis, piP-bodies generate MIWI2-piRNAs in preparation for their nuclear function, and the CB is specific for post-meiosis piRNA function. The functional relevance of processing piRNAs near mitochondria remains unclear, as well as the mechanisms that localize the piRNA precursors to IMC and the significance of CB formation for piRNA function.

## Developmental timing for piRNA production

Over 90% of the piRNAs in the adult testis are expressed during the pachytene stage of meiosis. Since these pachytene piRNAs are not required for the completion of meiosis, production at the pachytene stage, rather than a functional necessity, is more likely to be a biogenesis requirement, with the pachytene stage likely providing optimal conditions for the massive production of piRNAs. Meiosis prophase I can be divided into five phases based on chromosome behavior: leptotene, zygotene, pachytene, diplotene, and diakinesis. The pachytene stage, when paternal and maternal chromosomes undergo synapsis and pair with each other, takes the longest time, lasting over a week in mice. The formation of the synaptonemal complex, a ladder-like series of parallel threads that form between homologous chromosomes, is a hallmark of the pachytene stage(Li and Schimenti 2007; Reynolds et al. 2007). Synapsis in mice is coupled with double-strand break repair, resulting in crossing-over. Because male mammals are the heterogametic sex and thus have sex chromosomes of different sizes, the sex chromosomes in male spermatocytes cannot be completely synapsed. A process known as meiotic silencing of unsynapsed chromatin (MSUC) (Turner et al. 2006; Khalil et al. 2004) results in entire sex chromosomes in male spermatocytes being transcriptionally inactive during the pachytene stage. MSUC has two consequences for piRNA biogenesis. First, none of the pachytene piRNA loci reside on sex chromosomes (Li et al. 2013). Second, transcriptional inactivation during synapsis leads to a decoupling of translation and transcription. For example, phosphoglycerate kinase 2 (PGK-2) is transcribed at pachytene stage and is temporally translationally suppressed until the round spermatid stage (Geisinger et al. 2021; Fine et al. 2019; Jamsai et al. 2015; Gold et al. 1983). Thus, piRNA biogenesis factor mRNAs that are translated at the pachytene stage need to be sorted separately from mRNAs experiencing translation repression. While most meiosis research focuses on chromosomal behavior during meiosis prophase, little is known about how transcription, splicing, polyadenylation, RNA exportation, and translation of mRNAs are orchestrated to initiate, promote, and exit meiosis. Our recent studies indicates that ribosome recycling factors as well as NMD pathways are specifically inhibited at pachytene stage. Otherwise, these pathways would compete the substrates with the ribosome-guided piRNA biogenesis (Sun et al. 2021; Shum et al. 2016). It is thus possible the sophisticated temporospatial mRNA regulation that enables pachytene progression also facilitates pachytene piRNA production.

## Sexual dimorphism of piRNA pathways

In contrast to flies and zebrafish, where defective piRNA pathways results in sterility in both sexes (Ketting 2011), piRNA pathway disruption in mice only leads to sterile males, whereas females remain fertile (Carmell et al. 2007; Kuramochi-Miyagawa et al. 2004). This sexual dimorphism could be due to oocytes having a silencing mechanism mediated by endogenous siRNAs, which prevents TE activation (Tam et al. 2008; Watanabe et al. 2008). However, the activation of siRNAs in oocytes is rodent specific and not found in bovines nor humans, suggesting that this piRNA-independent defense mechanism is not present in oocytes of other mammalian species (Flemr et al. 2013; Rosenkranz et al. 2015). Moreover, compared to most other mammals with 4 PIWI genes, rodents lack *Piwil3*, which has been shown to be specifically expressed in oocytes in hamsters, bovines, and humans (Yang et al. 2019; Tan et al. 2020; Ishino et al. 2021), suggesting that the Piwil3-piRNA pathway is specifically missing in the rodent lineage. To test whether the sexual dimorphic requirement for a piRNA pathway is specific for the rodent lineage, three independent groups have disrupted the piRNA pathways in hamsters and revealed that piRNA pathways are indeed required for maintaining germline genome integrity in both sexes (Zhang et al. 2021; Hasuwa et al. 2021; Loubalova et al. 2021). Thus, rodents are likely to be an outlier with the siRNAs replacing the Piwil3-piRNAs in oocytes, and piRNA pathways are generally required for germ cells of both sexes in animals.

Nonetheless, piRNA pathways in the ovaries of humans, bovines, hamster, and macaques do show distinct differences from those in testes with regards to piRNA abundance, piRNA size, modification, piRNA-associated PIWI proteins, and the genomic origins of piRNA species (Rosenkranz et al. 2015; Ishino et al. 2021; Hasuwa et al. 2021; Zhang et al. 2021; Loubalova et al. 2021; Yang et al. 2019; Tan et al. 2020), arguing that piRNA pathways are influenced by the sex of the cell lineage. For instance, in hamsters, compared to testis piRNAs, oocyte piRNAs come from a distinct set of intergenic genomic loci whose transcription are not driven by A-MYB. Piwil3 in hamster oocytes bind to ~ 19 nt piRNAs without 2′-*O*-methyl modification, and the binding depends on the phosphorylation of Piwil3. In hamsters, while Piwil1 binds to ~ 29 nt piRNAs in testes, they bind to ~ 23 nt and ~ 29 nt piRNA in oocytes, and they only bind to ~ 23 nt piRNAs in 2-cell embryos. The developmentally dependent regulation of piRNA size contradicts the notion that the size of piRNAs is determined by the footprints of the PIWI protein. Therefore, further understanding the route of sexual dimorphism of piRNA pathways, either due to sex chromosome or sex hormone differences, will help to understand sex-dependent biogenic regulation and its impact on piRNA functions.

## The evolution of piRNA pathways

Pervasive adaptive evolution among piRNA pathway proteins has been reported in both insect and vertebrate lineages (Yi et al. 2014; Levine et al. 2016, 2012; Simkin et al. 2013; Palmer and Whybrow 2007; Obbard et al. 2009; Kolaczkowski et al. 2011). Two hypotheses have been proposed to explain the force driving this positive selection. The first hypothesis, an arms race with TEs, has been proposed since the discovery of piRNAs (Malone and Hanno 2009; Aravin et al. 2007a). As a prime embodiment of the Red Queen’s race, which originated from studies of host virus battles (Daugherty and Malik 2012), this hypothesis successfully explains why piRNA sequences need to rapidly adapt to keep up with ever-changing TE sequences but fails to provide a satisfactory explanation for the necessity of continuously changing the piRNA pathway proteins. While it is possible that piRNA biogenesis proteins have to constantly adapt to the life history of each recently invaded TE, such as driving the transcription of new piRNA precursors embedded with TEs or targeting new TE transcripts in a different subcellular location, as unlikely viruses, the TEs cannot “fight back” to repress piRNA machinery (Blumenstiel et al. 2016). The second hypothesis is adaptive evolution driven by the ongoing tension between TE silencing and off-target effects (known as genomic autoimmunity) (Blumenstiel et al. 2016). Similar tension has been observed between phage and CRISPR systems (Koonin and Yutin 2020). As the piRNAs have been shown to target mRNAs, off-targeting is the cost for robust TE suppression. The selection for specificity and sensitivity of TE suppression varies at different stages of TE invasion, with high TE activation favoring high specificity, while low TE expression favors sensitivity. Although most studies on the evolution of piRNA pathway proteins were performed on *Drosophila*, the same principles to suppress TEs and avoid autoimmunity would hold true for mammalians, as TE piRNAs share biogenic protein factors with pachytene piRNAs.

**Fig. 3:**
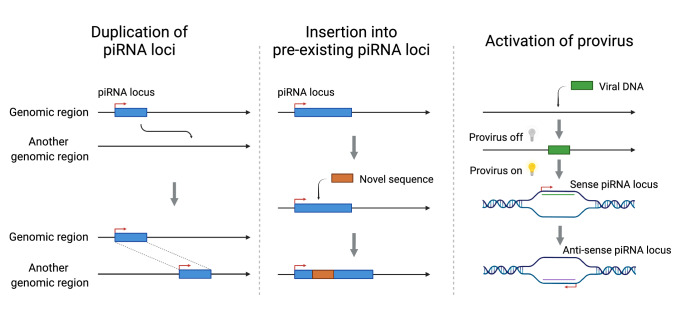
Current models of new piRNA acquisition. (1) Duplication of piRNA loci. piRNA clusters are duplicated or deleted in the genome and generate more piRNAs. (2) Insertion into pre-existing piRNA loci. New piRNAs can be generated by inserting sequences into pre-existing piRNA clusters. (3) Activation of a provirus for piRNA production. A provirus was first activated for piRNA production with sense orientation, and then the transcription template of the piRNA locus was switched. The direction of piRNA cluster transcription may change, and piRNAs can be generated from antisense piRNA loci. Figure created with BioRender.com

Pachytene piRNA sequences show poor conservation across species, and only 29 of 89 human piRNA loci share synteny conservation (the flanking genes surrounding piRNA clusters are conserved) outside of primates (Özata et al. 2020; Chirn et al. 2015). Despite being essential for male fertility, the function of pachytene piRNAs remains largely unknown. Although some pachytene piRNAs have been shown to trigger the decay of mRNAs required for meiosis or sperm maturation (Wu et al. 2020; Zhang et al. 2015; Gou et al. 2014; Goh et al. 2015), most pachytene piRNAs lack obvious complementary targets. It has also been proposed that PIWI proteins function without piRNAs (Vourekas et al. 2012), and piRNAs may exist to stabilize the PIWI protein without providing any specificity. Several models have been proposed to explain the rapid divergence of pachytene piRNA sequences. One hypothesis proposes that pachytene piRNA evolution drives reproductive isolation (Özata et al. 2020). Another suggests that existing piRNA clusters (Kawaoka et al. 2012) serve as landing pads for TE transposition ‘trapping’ new TE sequences in the cluster (Yamanaka et al. 2014; Zhang et al. 2020) (Fig. 3, middle), a mechanism reminiscent of the acquisition of new sequences in CRISPR loci. While it is known that piRNA loci can autonomously process transcripts harboring insertion sequences into new piRNAs (Muerdter et al. 2012), there is only limited evidence that TE transposition, or other insertion mechanisms, display a preference for targeting piRNA loci. Thus, the adaptive force underlying the rapid divergence of pachytene piRNA sequences currently remains unclear.

Two mutational modes are known to give rise to the birth of new piRNA loci. The first mode is copy number duplication or deletion of existing piRNA loci (Assis and Kondrashov 2009; Chirn et al. 2015) (Fig. 3, left). For example, mammalian piRNA loci have been reported to display elevated rates of copy number variation (Gould et al. 2012). However, it remains unclear what is the mutational mechanism leading to the copy number variations of piRNA loci. The second mode is activation of novel TE insertions into piRNA loci (Sun et al. 2017; Yu et al. 2019). For example, in koalas and chickens, proviruses that recently invaded the germline gained the ability to produce piRNAs. In koalas, piRNAs that target the KoRV-A retrovirus were originally produced from the sense strand of KoRV-A but then, through an unknown mechanism, shifted toward the production of piRNAs antisense to the retrovirus (Yu et al. 2019) (Fig. 3, right). Domesticated chickens were found to activate piRNA production from a truncated proviral locus against avian leukosis viruses, whereas undomesticated chickens did not produce piRNAs from the same locus (Sun et al. 2017). According to the “out of testis” hypothesis (Kaessmann 2010), the permissive chromatin state of spermatocytes and spermatids makes testes an ideal tissue for the emergence of new genes. Thus, it is not surprising that proviral loci are transcribed in testes, but it remains unclear what genomic and/or epigenetic mechanisms led to their ability to produce piRNAs.

## Perspective

piRNAs constitute a unique and rapidly evolving class of small non-coding RNAs with a wide diversity of functions and various biogenesis pathways. Developments in genetics and sequencing techniques have advanced piRNA research through the identification of multiple key players during the various phases of piRNA processing; however, the absence of cell culture systems, or in vitro systems, that can recapitulate mammalian piRNA production has hindered the mechanistic studies of piRNA biogenic pathways. Furthermore, studying mammalian piRNA function, especially pachytene piRNAs, using conventional genetics remains a challenge. As pachytene piRNAs share key piRNA biogenic factors with pre-pachytene piRNAs, animals with defective piRNA biogenesis exhibit de-silenced TEs that trigger arrest during early spermatogenesis, which consequently masks their function during spermiogenesis. Furthermore, A-MYB, the key pachytene piRNA transcription factor, also regulates the expression of (protein-coding) mRNAs such that *A-Myb* mutants also display early meiotic arrest (Li et al. 2013). Pachytene piRNAs are primarily derived from 100 intergenic genomic loci (Li et al. 2013) that are likely functionally redundant. As a result, deleting a single piRNA-producing locus often has no obvious phenotype (Wu et al. 2020; Homolka et al. 2015). Recently, sperm maturation defects were observed upon knockout of the promoter of a pair of piRNA clusters with only subtle changes of the entire transcriptome (Choi et al. 2021; Wu et al. 2020). In conclusion, significant advances have been made in our understanding of piRNA biology over the past several years; however, a number of key questions surrounding piRNA biogenesis remain:

1. Why are some transcripts processed to piRNAs while others are not? piRNA precursors could be either marked epigenetically during transcription or recognized post-transcriptionally in the cytosol. Unlike *Drosophila* germline piRNA loci, which are marked with the chromatin-bound protein Rhino (Klattenhoff et al. 2009), currently no epigenetic factors have been identified that specially bind piRNA loci in mammals. It is also unlikely that there would be unique splicing structures in mammals, as has been proposed for *Drosophila* piRNA biogenesis, because mammalian piRNA precursors are canonically spliced (Li et al. 2013; Sun et al. 2021). Whether pachytene piRNA precursors have unique secondary or tertiary structures, RNA modifications, or translation intermediates remains to be determined.
2. How are piRNA precursors recruited to mitochondria? What is the significance of the connection between piRNA biogenesis and mitochondrial biology? Either the piRNA precursors are channeled directly to the IMC from the nucleus without interacting with ribosomes prior to IMC localization or they are translated first, and the precursor translation intermediates are then transported to mitochondria. Given that the IMC is not close to the nucleus, if the former is the case, a special mechanism, such as packing piRNA precursors using RNA-binding proteins, would be required to prevent ribosome access prior to IMC localization. Alternatively, specific precursor translation intermediates could be recognized by the piRNA-processing machinery, or the translation products could be recognized by mitochondrion-localizing chaperones.
3. Is phased piRNA biogenesis initiated de novo or by initiator piRNAs? If phased biogenesis is triggered by base-pair complementary initiator piRNAs, the pairing rules between piRNAs and targets are likely to tolerant mismatches to allow sufficient cleavage events on the long single-stranded piRNA precursors. Alternatively, piRNA precursors may recruit an endonuclease or the conventional RNA decay machinery for de novo piRNA processing. One possibility is that piRNA biogenesis may be initiated by an endonuclease that specifically targets ribosomes outside of ORFs, as translation-mediated RNA decay is common, involving mechanisms such as NMD, no-go decay, no-stop decay, or co-translational Exo1-mediated decay (Shoemaker and Green 2012; Graille and Séraphin 2012; Kervestin and Jacobson 2012; Schoenberg and Maquat 2012).
4. How does the first nucleotide uridine (1U) bias arise? 1U bias is the most noticeable sequence signature on primary piRNAs. Crystal structures of the silkworm PIWI protein Siwi(Matsumoto et al. 2016) and *Drosophila* Piwi (Yamaguchi et al. 2020), as well as in vitro biochemical assays from silkworm(Matsumoto et al. 2016), revealed that the 5′-uridine fits well into the PIWI/Siwi MID domain. However, in *Pnldc1* mutant mice, the un-trimmed piRNAs form a head-to-tail pattern with no gaps between them, indicating that the fragmentation process must generate the 1U bias prior to PIWI loading. Since MitoPLD/ZUCCHINI does not show any preference for cleaving before uridines in vitro, nor do ribosomes display any binding bias, either MitoPLD is not the main endonuclease fragmenting the pachytene piRNA precursors (that show a 1U bias) or another co-factor exists that works coordinately with MitoPLD and ribosomes to generate the 1U bias.
5. Why are large quantities of piRNAs produced at the pachytene stage? We are yet to understand the biological significance of the burst of piRNA production at the pachytene stage. This production is due either to a functional or biogenic requirement. As discussed previously, while a biogenic requirement seems more likely, the disruption of pachytene piRNA biogenesis in hamsters leads to pachytene arrest (Hasuwa et al. 2021; Loubalova et al. 2021; Zhang et al. 2021), distinct from round spermatid arrest in mice, suggesting that pachytene piRNAs may be generally required for meiosis progression with rodent as an outlier. As most studies on meiosis have focused on chromosome behavior or epigenetic regulation, we know little about how RNA metabolism, such as translation and RNA granule movement, coordinates with the complex choreography occurring in nuclei. Such studies may shed light on the biogenic requirements that facilitate the coordinate production of 3.8–8.4 million piRNA molecules in each spermatocyte during the pachytene stage (Gainetdinov et al. 2018).
6. How is a new piRNA locus born and how does it die during evolution? Pachytene piRNA loci can rise and disappear rapidly over short evolutionary timescales (80 million years between mice and humans) in mammals. This rapid divergence could be due to elevated mutation rates and/or adaptive selection. The current notion that pachytene piRNAs function to target mRNAs required for sperm maturation does not seem sufficient to provide such a selective force. The idea that pachytene piRNAs and their mRNA targets behave like a toxicant and anti-toxicant is an attractive model that needs further investigation (Aravin 2020). The possibilities that piRNA loci exhibit elevated mutation rates or that piRNA loci represent preferential TE landing sites have also not been fully explored.
7. Are there somatic piRNAs in vertebrates? The notion is that somatic piRNA pathways widely exist in invertebrates, but was lost in vertebrates (Lewis et al. 2018). However, there are a number of reports in vertebrates regarding the existence of piRNA-like molecules or the expression of PIWI proteins (Galton et al. 2021; Mai et al. 2020; Moyano and Stefani 2015; Keam et al. 2014; Yan et al. 2011; Mei et al. 2015; Zhao et al. 2015; Nandi et al. 2016; Perera et al. 2019; Sharma et al. 2001; Freedman et al. 2016; Martinez et al. 2015; Lee et al. 2011; Cheng et al. 2014). These reports failed to provide a satisfactory answer as to why there is no phenotype beyond reproduction when disrupting piRNA pathways in mice. It is an interesting area for further investigation: either somatic piRNAs exist in low abundance with under-explored function, or mice are not a good model to study somatic piRNAs.

Here, we have provided an overview of piRNA biogenesis in mice and have referenced a range of other organisms where relevant. We discuss the current progress in our understanding of piRNA conservation and evolution. Finally, we outline several key questions in the field regarding piRNA biogenesis. With the rapid advances in sequencing technologies and development of new techniques and model organisms for multi-omics and comparative studies, we envision that significant strides will be made in the next few years. A complete understanding of piRNA biogenesis, evolution, and function may facilitate the development and application of artificial piRNAs as tools for epigenetic regulation and a wide variety of other possible uses.

Box 1 In 2002, well before piRNAs were discovered to bind to PIWI proteins and function as part of an RNA–protein complex, the Tetrahymena PIWI protein (Twi1p) was discovered to associate with small ~ 28 nt RNAs(Mochizuki et al. 2002) that were necessary for the elimination of internal eliminated sequences (IES) (Mochizuki et al. 2002), leading to the rearrangement of the Tetrahymena macronucleus genome (Jahn and Klobutcher 2002; Yao et al. 2003). In the absence of Twi1p (Twi1p knockout), cells failed to produce small RNAs and did not complete IES elimination. Mechanistically, these small RNAs function together with Twi1p to direct the elimination of IESs through both homology scanning and epigenetic regulation (Mochizuki and Gorovsky 2004; Mochizuki et al. 2002; Yao et al. 2003). Although these small RNAs are produced from double-strand RNAs via a Dicer-dependent pathway rather than from single-stranded RNA precursors, this RNAi-like mechanism provided the first hint that PIWI proteins function together with small RNAs now known as piRNAs. It also suggests that diverse RNA-processing mechanisms may converge on piRNAs.
